# Patient Experiences and Perspectives When MyChart is Introduced in a Large Community Hospital: Mixed Methods Study

**DOI:** 10.2196/66353

**Published:** 2025-01-23

**Authors:** Shelley Vanderhout, Shipra Taneja, Kamini Kalia, Walter P Wodchis, Terence Tang

**Affiliations:** 1 Institute for Better Health Trillium Health Partners Mississauga, ON Canada; 2 Institute of Health Policy, Management and Evaluation University of Toronto Toronto, ON Canada; 3 Trillium Health Partners Mississauga, ON Canada

**Keywords:** patient portal, implementation, mixed methods, patient experiences, patient perspectives, learning health systems, LHS, mixed methods study, electronic medical records, health information, caregiver experience, Canada, user satisfaction, descriptive statistics, thematic analysis

## Abstract

**Background:**

Patient portals, or secure websites linked to electronic medical records, have emerged as tools to provide patients with timely access to their health information. To support the potential benefits of patient portals such as improved engagement in health care, it is essential to understand how patients and caregivers experience these portals.

**Objective:**

This study aimed to explore patient and caregiver experiences, facilitators, and barriers to accessing and using a patient portal called MyChart during the initial stages of its implementation.

**Methods:**

We applied explanatory sequential mixed methods to conduct a web-based questionnaire and semistructured interviews with MyChart users and nonusers at a large community hospital in Ontario, Canada. Among users, we explored user satisfaction with MyChart, its impact on care, and areas for improvement. For nonusers, we explored barriers to MyChart access and willingness to use it in the future. Descriptive statistics and thematic analysis were used for data analysis.

**Results:**

A total of 5651 patients and caregivers completed the web-based questionnaire and 18 (12 users and 6 nonusers) participated in interviews. MyChart users primarily learned about the portal through email (n=1288, 39%), after-visit summaries (n=953, 29%), and hospital staff (n=408, 12%). Nonusers cited lack of awareness (n=1291, 59%) and registration difficulties (n=707, 32%) as some barriers to activation and adoption, but the majority would consider activating and using MyChart if they could learn more about it (n=1126, 54%). Users valued MyChart for preparing for health care encounters but expressed dissatisfaction with limited features and access to medical history and test results, whereas nonusers tended to be unsure about the benefits of using MyChart, especially if they were infrequent health care users.

**Conclusions:**

Patient portals offer benefits, but barriers to access and limited functionality can hinder widespread use. To enhance the adoption and potential benefits of patient portals, targeted outreach and comprehensive access to health information are essential to promote positive and seamlessly integrated health care experiences.

## Introduction

Patient portals are secure websites or mobile apps tethered to electronic health records that allow patients to view medical notes and test results, visit summaries, manage appointment schedules, and update personal information [1]. Designed to empower patients, they can promote patient engagement in care, increase self-management [2], improve patient-provider communication, and foster shared decision-making between patients and health care providers [3,4]. Portal use has led to improved communication and trust between patients and providers [5,6], adherence to care plans [7], and satisfaction with care [8]. Patient portals also have the potential to reduce anxiety about test results, avoid unnecessary visits, and improve health understanding [5,9,10]. However, patient portal uptake can be limited by low digital and health literacy [11,12], hesitancy about privacy and security of personal health information [13], and poor alignment of patient expectations for portal functionalities and features actually offered [12].

As health systems transition to electronic health records, many offer access to patient portals and have opportunities to learn from introducing these tools. Exploring patient experiences with accessing and using portals, barriers to activation and use, and perceived impacts on daily life and care is crucial to inform how portals can support high-quality care, identify barriers and enablers to their use, develop tailored supports for specific populations (eg, older patients), and highlight areas for improvement [14]. Particularly, when patient populations are diverse, health systems need to understand how patient portals may inadvertently exacerbate existing equity issues related to accessibility and technological literacy [15].

This study was an evaluation to generate transferable knowledge about the implementation of a patient portal called MyChart within the first 6 months of its launch at a large community hospital in Ontario, Canada. Specifically, we sought to answer the following questions:

- Among patients who activated MyChart, what were their initial experiences, perceived impacts, and suggestions for improvement?
- Among patients who did not activate MyChart, what were the barriers to doing so, and what were their preferences for support to access and use MyChart?
- How can health systems offer portals that meet patients’ needs and expectations?

## Methods

### Setting

This mixed methods evaluation took place at Trillium Health Partners (THP), a large community hospital comprised of 3 sites in Mississauga, Canada. Mississauga is a very diverse community in Canada with 51% of residents identifying as newcomers and 62% as visible minorities. THP operates 1457 inpatient beds and has more than 1.7 million annual patient visits (including 225,723 emergency and urgent care visits and 776,308 outpatient visits), with more than 11,000 staff and physicians [16].

THP implemented the Epic electronic health record in October 2020, and deployed Epic’s patient portal, MyChart, in September 2023 [17]. At THP, patients can activate MyChart on a desktop computer or through the MyChart mobile app through links to create an account included in reminder emails about upcoming visits, unique codes provided on after-visit or discharge summaries, or in-person during a care encounter. THP offers technical support for MyChart via telephone and email, tip sheets, and instructional videos on their website about activating accounts and using the available features. MyChart is advertised through posters in hospital waiting rooms and corridors, social media, the THP website, and word of mouth (eg, clinical clerks during visit check-in and health care provider champions who encouraged their peers to socialize MyChart with patients). At THP, MyChart functionalities were introduced in a staged manner with new features launching every quarter; the first stage included viewing after-visit and discharge summaries, outpatient test results, and viewing or updating personal information, medications, allergies, and vaccinations. The second stage added viewing appointment schedules, electronically checking in for or canceling appointments, launching video visits, and requesting additional information. Messaging between patients and providers was not offered as part of MyChart’s launch at THP. This approach was taken to reflect organizational capacity and early engagement with health care providers that suggested limited readiness for comprehensive features to be introduced all at once, especially messaging.

### Approach

We applied an explanatory sequential mixed methods evaluation approach to understand patient and caregiver experiences, both among those who had activated MyChart and those who had not [18]. This mixed methods evaluation included: (1) a web-based questionnaire of MyChart users and nonusers with different questions for each group and (2) semistructured interviews with users and nonusers to add richness and depth to the questionnaire data. The RE-AIM Implementation Framework [19] and its extension [20] to enhance sustainability and promote equity guided our approach and informed questionnaire and interview content. This framework allowed us to focus on key areas such as reach (identifying users and barriers), effectiveness (assessing patient-perceived impacts and potential negative outcomes), adoption (examining use patterns and disparities), implementation (identifying effective facilitators), and maintenance (evaluating long-term impact).

### Data Collection and Analysis

We invited all THP patients to participate in a web-based questionnaire (Multimedia Appendix 1), hosted on Qualtrics for 4 weeks during March 2024 [21]. We explored users’ satisfaction with MyChart, perceived impacts on care, and suggestions for improvement. For nonusers, we investigated barriers and potential enablers to activating MyChart, and alternative ways they would like to be involved in their care. Participants had the option to provide their age, gender identity, ethnicity, and postal code to help characterize user and nonuser populations. The questionnaire included both closed- and open-ended questions and was created based on available literature [9,13,22,23] and peer health systems’ evaluations. It was revised based on feedback from a MyChart patient advisory council at THP.

All patients with an email address recorded and who had provided consent to receive nonclinical communication were invited by email to participate; caregivers were able to participate if their email was linked to a patient’s record. Two reminder emails were sent 3 and 5 days following the initial invitation. We advertised the questionnaire on the hospital’s website, social media pages, and posters with QR codes in waiting areas. MyChart users saw a banner with a link to the questionnaire upon signing into the platform. Acknowledging that not all patients would be reached virtually, hospital volunteers also recruited patients to complete the questionnaire on tablets from clinic waiting rooms and offered navigational support when necessary. The questionnaire took approximately 5 minutes to complete, and participants were not offered incentives. At the end of the questionnaire, participants could indicate if they would like to participate in a follow-up interview.

Descriptive statistics were used to summarize responses to closed-ended questions and participant demographics, and thematic analysis was used to identify key concepts among answers to the open-ended questions. The Ontario Marginalization Index (ON-Marg) was used to classify postal codes according to socioeconomic status. ON-Marg assigns quintiles to postal codes based on relative material deprivation (representing education, low income, unemployment, lone-parent families, and dwellings in need of major repair) and ethnic concentration (representing immigrants within the past 5 years and visible minorities) [8]. Participants were excluded if they indicated that did not receive care at THP in the questionnaire. Statistical analysis was completed on SAS (version 9.4; SAS Institute).

We used a combination of purposive sampling and maximum variation to invite 18 questionnaire participants to individual semistructured interviews while seeking variability in age, ethnicity, gender, and overall sentiment of questionnaire response [24]. Separate interview guides were used for MyChart users and nonusers (Multimedia Appendix 1). All interviews were conducted by one team member (ST) via Zoom (Zoom Communications Inc), lasted approximately 30 minutes each, and were recorded and transcribed verbatim. Transcripts were reviewed by the interviewer to verify completeness and accuracy and were not reviewed by the participant. Two members of the research team (ST and SV) conducted thematic analysis on NVIVO (version 12; Lumivero); following data familiarization, they conducted an initial coding phase, combining a deductive and inductive approach. Deductive coding was based on the RE-AIM framework [19], which guided the identification of text related to reach, effectiveness, adoption, implementation, and maintenance indicators, as well as the acceptability and accessibility of MyChart. Inductive coding was used to explore patients’ experiences and perspectives. Codes were used to generate themes, each built around a central organizing concept, which were then refined iteratively to produce a final set of themes.

### Ethical Considerations

This study was reviewed by the THP Research Ethics Board and classified as quality improvement. This study was classified as quality improvement as it aimed to enhance existing processes within our health care system. All participants provided informed consent prior to participating in the questionnaire and interviews. They were informed that the study results would be deidentified and published in peer-reviewed journals and potentially shared with the broader public. Interview participants were given a CAD $30 (US $21) honorarium for their time.

## Results

### Overview

In total, 5651 patients and caregivers completed the questionnaire. Of those 5651 participants, 3336 (59%) participants were MyChart users and 2315 (41%) participants were nonusers, and most of their characteristics were similar across groups except for age; younger individuals were less represented among MyChart users (Table 1). Of the MyChart users, 3098 (93%) participants used MyChart for their own care, and 417 (13%) participants used it as a parent or caregiver. A total of 18 patients and caregivers participated in interviews; twelve (67%) were MyChart users and 6 (33%) were nonusers. Three MyChart users interviewed were parents or caregivers, and 2 used MyChart as users and as family members.

Most MyChart users learned about the patient portal through various channels, including hospital emails (1288/3336, 39%), after-visit summaries (953/3336, 29%), their care team (935/3336, 28%), hospital staff (408/3336, 12%), informational posters (340/3336, 10%), knowledge of MyChart at other health care systems (163/3336, 5%), through recommendations from family or friends (125/3336, 4%), or social media (60/3336, 2%). While 775 (34%) of nonusers were aware of MyChart and expressed interest in learning more, the most reported barrier was a lack of awareness that it was offered (1291/2315, 59%). Other commonly reported barriers included uncertainty about how to sign up for an account (707/2315, 32%) or use the portal (516/2315, 23%). A smaller proportion of nonusers reported low digital literacy (322/2315, 15%) or concerns about privacy and security (267/2315, 12%) as barriers to activating MyChart. Nonusers indicated that they would consider activating and using MyChart if they had the opportunity to learn more about its features (1126/2315, 54%), and how to create an account (968/2315, 47%). Web-based videos were the most preferable method for learning (943/2315, 62%).

**Table 1:** Questionnaire and interview participant characteristics.

Characteristics^a^			Users (n=3336), n (%)		Nonusers (n=2315), n (%)		Interviewed users (n=12), n (%)		Interviewed nonusers (n=6), n (%)
**Age (in years)**									
	12-20	1 (0.4)		18 (0.9)		0 (0)		0 (0)	
	21-40	262 (9)		282 (14)		1 (8)		1 (17)	
	41-60	964 (33)		625 (32)		6 (50)		2 (33)	
	61-80	1653 (57)		943 (48)		5 (42)		2 (33)	
	≥81	119 (4)		114 (6)		0 (0)		0 (0)	
**Self-identified gender**									
	Man	1128 (36)		743 (36)		3 (25)		0 (0)	
	Woman	1951 (62)		1281 (62)		9 (75)		6 (100)	
	Prefer not to answer	68 (2)		44 (2)		0 (0)		0 (0)	
	Other	9 (0.3)		9 (0.4)		0 (0)		0 (0)	
**Cultural and racial background^b^**									
	White	1965 (63)		1245 (61)		6 (50)		2 (33)	
	South Asian	375 (12)		272 (14)		3 (25)		2 (33)	
	East Asian	169 (5)		83 (4)		1 (8)		1 (17)	
	Southeast Asian	164 (5)		85 (4)		0 (0)		0 (0)	
	Black	132 (4)		116 (6)		1 (8)		1 (17)	
	Middle Eastern	86 (3)		69 (3)		0 (0)		0 (0)	
	Latin American	68 (2)		57 (3)		0 (0)		0 (0)	
	First Nation	6 (0.2)		6 (0.3)		0 (0)		0 (0)	
	Métis	4 (0.1)		6 (0.3)		0 (0)		0 (0)	
	Indigenous/Aboriginal	2 (0.1)		6 (0.3)		0 (0)		0 (0)	
	Inuit	0 (0)		1 (0.05)		0 (0)		0 (0)	
	Prefer not to answer	123 (4)		107 (5)		0 (0)		0 (0)	
	Other	59 (1.9)		32 (2)		0 (0)		0 (0)	
**Neighborhood material resources, quintile (%)**									
	1=lowest neighborhood material resources	700 (24)		457 (24)		3 (25)		0 (0)	
	2	839 (29)		510 (27)		4 (33)		2 (33)	
	3	731 (25)		448 (24)		4 (33)		2 (33)	
	4	427 (15)		301 (16)		1 (8)		1 (17)	
	5=highest neighborhood material resources	224 (8)		154 (8)		0 (0)		1 (17)	
	Unmatched to ON-Marg^c^	18 (0.6)		24 (1.3)					
**Racialized and newcomer populations, quintile (%)^d^**									
	1=lowest neighborhood proportion of racialized and newcomer populations	80 (3)		41 (2.2)		1 (8)			
	2	223 (8)		186 (10)		0 (0)		1 (17)	
	3	524 (18)		362 (19)		2 (17)		1 (17)	
	4	858 (29)		520 (27)		7 (58)		1 (17)	
	5= highest neighborhood proportion of racialized and newcomer populations	1236 (42)		761 (40)		2 (17)		3 (50)	
	Unmatched to ON-Marg	18 (0.6)		24 (1.3)					

^a^Characteristics were optional questions.

^b^Participants could select multiple options.

^c^ON-Marg: Ontario Marginalization Index.

^d^Data only available for postal codes that could be matched with ON-Marg.

**Figure 1:**
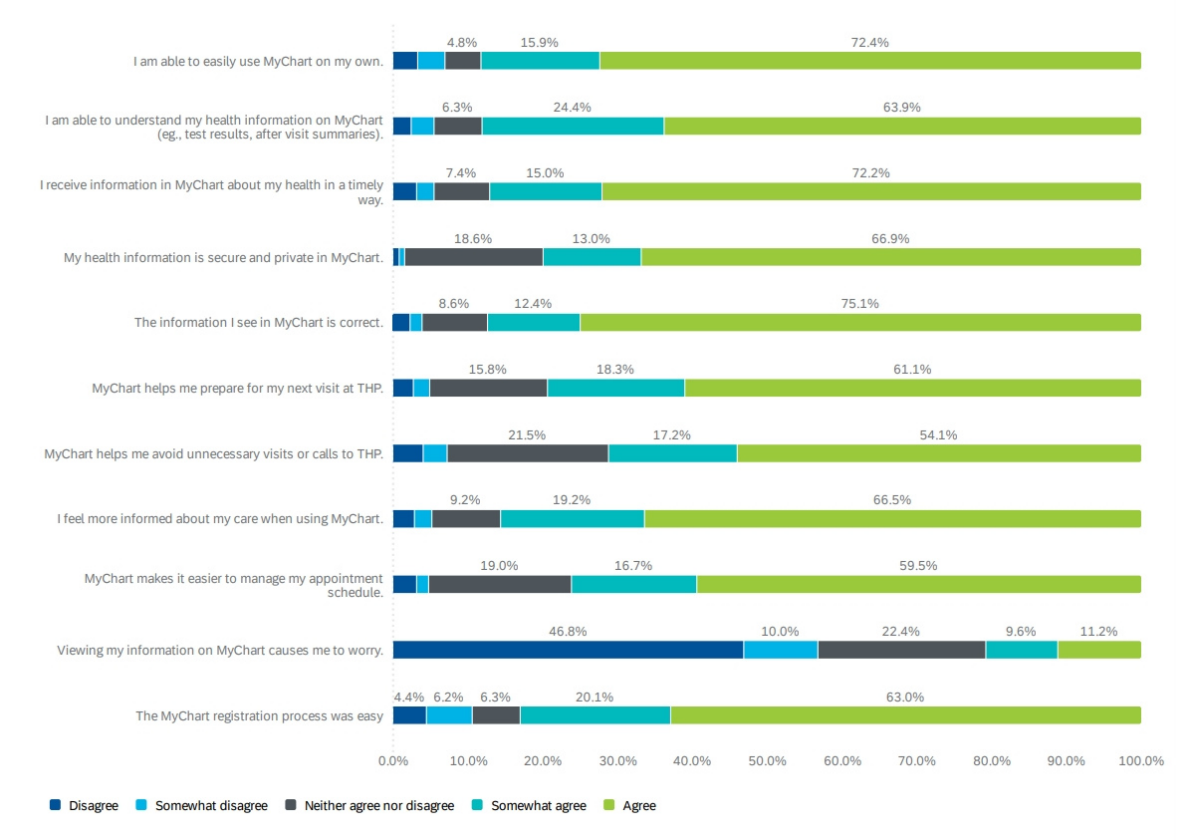
User perspectives about using MyChart. THP: Trillium Health Partners.

Perspectives of MyChart users about the portal are shown in Figure 1. Most MyChart users who participated in the questionnaire agreed or somewhat agreed that the portal was easy to use independently (2907/3336, 88%), they could understand the information available in MyChart (2897/3336, 88%), and they felt more informed about their care with MyChart use (2744/3336, 85%). Similarly, 79% (2558/3336) of users agreed or somewhat agreed that MyChart helped them prepare for health care encounters. When asked whether accessing their information caused worry, there were varying responses; over half of the participants (1804/3336, 57%) disagreed or somewhat disagreed that MyChart use caused any worry while 663 (21%) participants agreed or somewhat agreed that it did cause worry. A total of 80% (2605/3336) of users agreed or somewhat agreed that their personal health information was secure and private in MyChart.

Thematic analysis of responses to open-ended questionnaire items and interview questions produced 2 main themes, which are summarized below.

### Access to Information

Patients and family members using MyChart praised the platform for its user-friendly interface and convenient access to health information. They appreciated the right to review their own health records and found it empowering to be informed about their health care. A few patients described the feature of revisiting past notes from their care team to be particularly helpful with information recall.

And so what I have found is MyChart has been very helpful for me in providing one spot for me to know, what are my upcoming appointments at this hospital, what test results are there that I can refer back to if another doctor asks me did I do this or what was this or how is this looking. And at least giving me some insight and awareness into what’s happening and not having to wait for a doctor’s appointment to get answers to questions I have.

User 2

One nonuser who had an interest in MyChart described how beneficial it could be for caregivers who could not attend appointments, as it offered a window into the discussion and decisions made, fostering a better understanding of their loved one’s care.

It’d be interesting to see if you can integrate notes or directions on what to do when you get home and that kind of thing so that when you have the elderly going in ... he has his phone. My in-law is in his late 60’s. He has an iPhone. To have that integrated for him so when me or my husband go back and we look at the notes we’re like, OK this is what happened when he went. Then we can go and say ... I don’t have to take time off work, we can understand the notes, translate it for him and then go back and go, OK, this is what you need to do now. Or this is what you have to look out for versus having to take the time off work, go with him to literally be there. That would be interesting, I don’t know if that’s a feature or a future feature.

Nonuser 4

While patients and caregivers appreciated access to MyChart, they perceived the available features and functionalities at THP to be very limited, especially relative to other health systems. They desired access to a wider range of information, including consultation notes, medical images, emergency department results, and a more comprehensive medical history. After-visit summaries were available on MyChart, and while accessible, were seen as lacking detail (since they did not include health care providers’ chart notes from the visit) and were often outdated, hindering patients’ understanding of their care plan.

One thing that would be really nice to have is access to the doctor’s notes ... like, the notes from your appointment. I tend to try to take my own notes when I’m at clinical appointments but sometimes it’s hard because I’m trying to be present in the appointment and listen and try to understand in the moment. Sometimes my recall isn’t great and my note-taking isn’t great. So having access to a doctor’s summary of what it is that they’ve talked about and the direction and guidance that they’ve given would be helpful to be able to go back to and refer to, and even just to update my own notes. So, that’s another thing because right now that is not something that I see or that’s accessible in terms of their note. But I know it gets documented and shared with other providers in my circle of care but I don’t get to see that. And so sometimes it’s hard. It’s ... like, that would be great. It would be great if that was accessible.

User 2

Beyond limited information, patients and caregivers identified issues with MyChart’s lack of content and functionality. They envisioned MyChart bridging the gap between care providers but felt it operated in isolation, hindering its full potential.

There’s no connectivity. The whole thing is to streamline the process and to make sure that the patient has access to their records ... So, if that’s not being sent to the technology, to the app, then I’m not getting the information. So, what’s the use of spending all this money on technology that is not being used to its fullest?

User 4

Among current users, there was a desire for additional features such as chat or messaging with providers (n=476), access to more information such as imaging, historical information and notes (n=550), sending records or providing access to family members or proxies (n=169), linking to other hospitals and care providers (n=271), and supporting information for interpreting results (n=111). Some of these features were in fact available to MyChart users, such as adding proxy users and high-quality resources to help understand medical information, but this finding highlighted low awareness of MyChart’s full range of functionalities.

Access to medical records and test results presented a double-edged sword for both users and nonusers. Some users saw it as a source of comfort, reducing anxiety by eliminating the stressful waiting periods and uncertainty before appointments; viewing results ahead of appointments allowed them time to process information and prepare questions for follow-up visits.

For example, my family doctor suspects there’s something wrong. Sends for a biopsy. The biopsy results are back. Good or bad. Most of the time let’s say it’s good. But you have that two weeks of stress that you didn’t really need. And let’s say it’s negative. It’s still better to know that you have cancer and how to deal with it because you’ve got time to process. So, by the time you go to the doctor you’re prepared to ask the questions that need to be asked. So, it’s a more productive meeting between the patient and the doctor and it alleviates the stress level.

User 4

However, nonusers expressed that immediate access to sensitive test results before appointments would increase their anxiety. While some patients and families may have medical knowledge to interpret the notes, nonusers described it as a “folder of anxiety” as it does not contain context and next steps provided by a health care provider.

So if we could see the results and know we were talking to our doctor either on phone or in person, within 24 hours, we would sign up for MyChart, I guess is the most simple ... When you’re talking hypertension, cholesterol, thyroid. I guess, like, regular run-of-the-mill stuff. Then fine. Like, I know what’s going to happen. You’re going to adjust my dose. You’re going to add another medication. You’re going to, like, whatever it is. When you’re talking about life-threatening and life-changing things, like everything changes.

Nonuser 1

### Barriers to Activation and Adoption of MyChart

Despite MyChart’s potential benefits, patients and caregivers who were aware of MyChart but had not activated accounts shared hesitations about signing up. This included difficulty navigating the registration process, misperceptions about the cost to activate an account, a perceived low need for MyChart since they were infrequent health care users or felt the available features were too limited, and a preference for face-to-face interactions. The registration process was a significant challenge for both MyChart users and nonusers, as they attempted to register MyChart accounts but faced roadblocks; despite some patients trying to seek assistance from the hospital or family members, a few encountered difficulties such as 2-factor authentication, which combined with their limited technical skills, ultimately led them to abandon the registration process.

I have to put all these numbers, whatever it is and then they say it’s not right. Again, I’m putting in the numbers, it’s not right. I did it three times, after that I think it says your time has expired so come back. So, how many times am I going to put in the numbers and still I’m not getting anyplace? That is where my frustration starts. One time, two times, three times. It’s hard to understand why they have to think that every ... I know the younger generation picks it up very fast. Why do they think that everybody is that fast, especially the seniors.

Nonuser 3

Some nonusers who were accustomed to advanced features and interconnectedness in other apps found MyChart underwhelming. They questioned the effort involved in registration and some were frustrated by the fragmentation of separate patient portals offered by different providers (eg, family physicians and physiotherapists). This fragmentation hinders information flow, creates repetitive tasks to create accounts and add personal information, and forces patients to manually connect the dots between health care providers.

But in addition to my doctor, everybody I’ve been to has one of these portals, and they’re all different. If you had all signed up for the same premade system, I’d be willing to learn it in order to talk to all the medical people in my life ... So the thing is it adds up and it adds up and they’re all different. And the thing is as a patient you might only use it two or three times and never see that specialist again.

Nonuser 2

Some infrequent health care users mentioned they did not need features like record keeping and checking medical results and felt comfortable receiving information through face-to-face interactions, especially if they lacked the health literacy required to understand the information presented in MyChart.

[...] It’s no use to me if I’m not talking to the doctor about it ... I get the general drift of things because I know medical vocabulary and I work in that area. But I don’t have the knowledge of a doctor. And without that you can go very far astray.

Nonuser 2

## Discussion

### Principal Results

This mixed methods study explored the initial experiences and perspectives of 5651 patients about MyChart implementation at a large community hospital, including facilitators and barriers to accessing and using the portal. While most patients learned about MyChart through postvisit summaries, hospital staff, and email communications, barriers such as limited awareness, misconception around cost, registration difficulties, and unclear benefits of the portal hindered its uptake among nonusers. Users generally perceived MyChart to be user-friendly and enhance their ability to understand and prepare for their care. However, both users and nonusers highlighted the importance of a portal meeting their needs and expectations as a condition for uptake and impact on their care. Many participants highlighted a desire for more functionalities, such as access to imaging results, consultation notes, messaging with providers, and tools for interpreting medical information, which was often driven by comparisons to patient portals at other hospitals that offered more features. Expectations also included connectivity across a range of providers and institutions, frictionless account set-up and access, and technical support. Nonusers expressed interest in learning more about MyChart and its specific functionalities through educational materials to help them better understand the portal and its benefits, as well as technical and registration support to facilitate easier access.

### Comparison With Prior Work

Our study participants consistently highlighted MyChart as a valuable tool for accessing timely health information, preparing for visits, managing appointment schedules, and actively participating in their health care. However, whether they were MyChart users or not, participants in this study identified several barriers to accessing the patient portal, including a lack of knowledge of the platform, registration difficulties, limited interest in available features, a preference for face-to-face interactions, and low digital literacy. MyChart users were more often older individuals, which may reflect an increase in health care use with age and a higher need for managing personal health information. Notably, a similar proportion of users and nonusers resided in neighborhoods characterized by racialized or newcomer populations, or those with low material resources, which is inconsistent with use patterns observed in other studies—often, portal users have higher socioeconomic status than nonusers [14,25,26]. Our findings may be reflective of the patient population in our health system’s catchment area, which has a high proportion of newcomer and lower-resourced groups. In both our study and the literature, access to medical records and clinical notes can be beneficial for marginalized populations in understanding and managing their own care, as it may help overcome challenges to do with language, digital literacy, and recalling information, for example [27].

In this study, immediate access to medical records and test results was a complex issue. The majority of MyChart users believed this access empowered them with greater control and understanding of their health information and lessened anxiety associated with waiting for test results. Conversely, nonusers worried about potential misunderstandings and negative outcomes of viewing concerning medical information, especially before they could discuss it with a health care provider. Numerous studies in the literature [28,29] align with these perspectives, indicating that patients appreciate timely information but there is potential for negative emotions. From our findings, it is unclear whether nonusers only perceive the risk of higher worry, or if self-awareness of anxious tendencies causes certain patients to avoid portals altogether. Health and digital literacy may also differ among MyChart users and nonusers, where those who feel more equipped to understand health information and use other tools such as credible health websites to interpret complex details may be more likely to use MyChart [30]. Some research [29] revealed a strong patient preference for receiving test results through a portal regardless of whether they were normal or abnormal; however, health care providers have expressed concerns about this practice [28,31,32], suggesting increased patient anxiety and workload changes due to potential increases in messages, calls, and urgent visits to discuss the results. Many health care providers have advocated for a more structured approach, suggesting that patients should receive access to medical records and test results postappointment, allowing for discussion and clarification of results in a clinical context. However, this contradicts the strong preference MyChart users have for access to their complete health information as soon as possible, even if there is a risk of viewing concerning results without the immediate guidance of a health care provider, and may cause strain on health care providers to review and release results to patients within certain timeframes [2,28,29].

### Strengths and Limitations

This study examined the early experiences of patient portal implementation among users and nonusers at a large community hospital in Canada that serves a diverse population. With a strong response rate of over 5000 patients and caregivers, this study captures the experiences and perspectives of a wide range of health users. The use of a mixed methods design that combined both qualitative and quantitative approaches enabled us to understand the facilitators and barriers associated with portal adoption and use. By capturing the perspectives of both users and nonusers with different ethnicities and socioeconomic backgrounds, we generated a comprehensive understanding of the factors that influenced patient portal adoption and engagement. These rich insights improve the transferability of our findings across patient populations and health system settings and can inform a range of patient portal implementation strategies.

This study has limitations. While we used multiple recruitment strategies including in-person invitations, posters, and email communications, this study was limited to English speakers due to the language requirements of the questionnaire and interviews. Given this, the findings may not reflect all the views and perspectives of those in the Mississauga and West Toronto region and may not apply to other linguistically diverse populations. We also were not able to determine the exact number and characteristics of patients who were invited to participate using our multipronged recruitment strategy, which limited our ability to identify response rates among specific patient populations (eg, newcomer groups). As this study examined a staged roll-out of MyChart features, the results may not be applicable to other health care organizations using different implementation approaches. Finally, given that this study was about MyChart, our sample of nonusers may not have been fully representative of that population if the questionnaire was perceived to be less relevant to their experiences.

### Conclusions

In conclusion, our findings reveal actionable and transferable learnings about the implementation of MyChart. Low digital literacy and lack of awareness of MyChart’s available features were barriers to MyChart activation and use among both users and nonusers. A surprising barrier to accessing MyChart was a misperception about the costs associated with creating an account; given that several digital health tools require payment, health systems should clearly communicate when available portals are free to maximize uptake. Widely marketed and accessible interventions to support digital literacy and help patients learn about and navigate MyChart could increase patient awareness of available features and their potential to positively impact patient experiences. Similarly, improving patients’ health literacy could reduce perceived worry associated with viewing concerning results in MyChart, and drive additional benefits of using the portal by increasing the interpretability of information. This study highlighted a strong desire among some patients and caregivers for access to additional medical records and test results, and to exchange messages with health care providers. While expanding access to these features can improve patient engagement and care management, it is crucial to consider the potential impact on patient-provider relationships and provider workflows. At the same time, some patients had little interest in MyChart, which is important for health systems to consider when setting targets for patient portal uptake. As health systems increasingly offer patient portals to increase patient and family engagement in care, support shared decision-making, and provide patients access to their health information, a culture of timely, comprehensive access to complete medical records is building. To ensure uptake and positive experiences with portals, health systems need to meet patient expectations by providing access to a variety of features, especially when multiple health systems offer portals to the same population.

## Appendix

Multimedia Appendix 1 Questionnaire and interview guide.

## Abbreviations

ON-Marg: Ontario Marginalization Index
THP: Trillium Health Partners

## Data Availability

The datasets generated during or analyzed during this study are not publicly available as participants did not consent to share the data outside of the research team.
