# Supplementary material for: Patient Experiences and Perspectives When MyChart is Introduced in a Large Community Hospital: Mixed Methods Study
Source: J Med Internet Res. 2025 Jan 23;27:e66353. doi: 10.2196/66353 (PMC11803326; doi:10.2196/66353)
Supplement: Multimedia Appendix 1 [file jmir_v27i1e66353_app1.docx]

MyChart Questionnaire

1. I...
   1. Receive care at Trillium Health Partners OR Am a parent or caregiver for someone who receives care at Trillium Health Partners
   2. Do not receive care at Trillium Health Partners, and am not a parent or caregiver or someone who does -> EXIT SURVEY

*If 1a = Yes,*

1. Please select the most appropriate option
   1. I use MyChart for my own care OR as a patient/caregiver/other
   2. I do not use MyChart

*If 2a = Checked*

1. Please select all that apply
   1. I use MyChart for my own care
   2. I use MyChart as a parent/caregiver/other
2. I learned about MyChart through:
   1. My care team
   2. A clinical clerk
   3. An After Visit Summary received following an appointment
   4. Social media/the internet
   5. Email from the hospital
   6. A poster in the hospital/clinic
   7. A friend or family member
   8. Another hospital where I receive care
   9. Other (free text)

1. Please select the option that is most appropriate [disagree/somewhat disagree/neither agree nor disagree/somewhat agree/agree]
   1. The MyChart registration process was easy.
   2. I am able to easily use MyChart on my own.
   3. I am able to understand my health information on MyChart (e.g., test results, after visit summaries).
   4. I receive information in MyChart about my health in a timely way.
   5. My health information is secure and private in MyChart.
   6. The information I see in MyChart is correct.
   7. MyChart helps me prepare for my next visit.
   8. MyChart helps me avoid unnecessary visits or calls to THP.
   9. I feel more informed about my care when using MyChart.
   10. MyChart makes it easier to manage my appointment schedule.
   11. Viewing my information on MyChart causes me to worry.
2. I want to be able to do ___ (e.g., talk to my provider, send records to family members) through MyChart: free text
3. I have suggestions about next steps or improvements to MyChart: free text
4. Is there anything else you would like to tell us about your experience with MyChart?: free text

*If 2b = Checked*

1. Before today, I knew that MyChart was available at Trillium Health Partners.
   1. Yes
   2. No
2. I want to learn more about MyChart.
3. Yes
4. No
5. I know how to sign up for MyChart.
6. Yes
7. No
8. I know the purpose of MyChart.
9. Yes
10. No

For the following questions, select all that apply:

1. I have not signed up for MyChart because:
   1. I did not know about it.
   2. I’m not sure what the purpose of MyChart is.
   3. I’m not sure how to sign up for MyChart.
   4. I’m not sure how to use MyChart.
   5. I’m not good with technology.
   6. I don’t need the features MyChart offers (for example, accessing my medical results or managing my appointment schedule).
   7. I don’t have support at home (for example, from a caregiver) to use MyChart.
   8. I’m worried about the privacy and security of MyChart.
   9. I don’t have access to the internet.
   10. I don’t have a mobile device or computer.
   11. MyChart is not offered in my preferred language.
       1. Type preferred language
   12. Other: free text
2. I might sign up for MyChart if:
3. I can learn more about what it is and what it is used for.
4. I can learn how to sign up and use it.
5. I can access a mobile device or computer.
6. My health care provider encouraged me to do so.
7. MyChart was offered in my preferred language. (Please provide your preferred language)
8. None of the above, I don’t want to sign up.
9. Other: free text
10. How would you like to learn more about MyChart
    1. Videos on the internet
    2. One-on-one support in the hospital/clinic
    3. Virtual group information session (for example, on Zoom)
    4. In-person group information sessions
    5. Written materials
    6. Other: free text

*Demographics – All Participants*

This information is being collected so that we can better understand how to provide the best care for all members of the Trillium Health Partners community. If you choose to answer the questions below, we will not be able to identify you or link your responses to this questionnaire back to your identity. All the information collected in this questionnaire is private and will be stored on THP’s secure server.

1. Age
2. Gender identity
3. Genderfluid or genderqueer
4. Man
5. Nonbinary
6. Transgender man
7. Transgender woman
8. Two-Spirit
9. Woman
10. Questioning or unsure
11. Another gender identity (please specify): ______________
12. Prefer not to answer
13. People living in Canada come from many different cultural and racial backgrounds. The following question will help us to better understand the experiences of the communities that we serve. Do you consider yourself to be . . .
14. First Nation
15. Inuit
16. Métis
17. Indigenous/Aboriginal
18. Middle Eastern
19. Black (North American, Caribbean, African, etc.)
20. East Asian (Chinese, Japanese, Korean)
21. Latin American
22. South Asian (East Indian, Pakistani, Sri Lankan, etc.)
23. Southeast Asian (Vietnamese, Cambodian, Filipino Malaysian, Laotian, etc.)
24. White (North American, European, etc.)
25. Other (please specify)
26. Prefer not to answer
27. Postal code

*Optional MyChart Interview*

We would like to hear more about your experiences in a short (up to 30 minute) interview.

If you would like to learn more about participating in an interview about MyChart, please provide your contact information, and we may follow up with you if you are eligible.

We would be delighted if you could kindly share your Name, Email, and Phone Number with us.

One of our team members will reach out to you to arrange a date and time that aligns with your schedule. Your cooperation is greatly appreciated, and we look forward to speaking with you!

Interview Guide

By verbally consenting to participate in this study, you confirm that:

- This study has been fully explained to you and all of your questions answered to your satisfaction
- You understand the requirements of participating in this study
- You have been informed of the risks and benefits, if any, of participating in this study
- You have been informed of any alternatives to participating in this study
- You have been informed of the rights of participants
- You have read and reviewed the information letter provided to you
- You have agreed to participate in this study

Do you agree with these statements and consent to participate in the interview?

Thank you so much for being willing to share some of your experiences with us.

*MyChart Users*

[*Explain that the interview is confidential and remind the participant that their confidentiality and their right to withdraw consent will be respected at all times. Remind the participant that they can ask for a break at any time if they would like one. Explain that the interview will help the research team better understand how we can improve how MyChart works at THP.*]

1. Can you tell me about a time you used MyChart recently? How did this change your experience at THP from when you weren’t using MyChart?
2. What do you like the most about MyChart?
3. What do you like the least about MyChart?
4. Have you received the support you need to use MyChart?
5. Do you find it easy to navigate and use MyChart? Are there any specific features that are confusing or difficult to use?
6. Probes: login process, access from different devices, printing, sharing information with family/other providers
7. Do you feel that the information displayed in MyChart is accurate and up-to-date? Have there been any instances where you noticed inconsistencies or errors?
8. Do you find that using MyChart makes you more or less anxious about navigating your health experience? How so?
9. In terms of privacy and security, do you feel that your personal health information is adequately protected within the MyChart platform?
10. If we could improve MyChart or add features to it, what would you suggest?
11. Is there anything else you’d like to tell me that we didn’t cover today?

Thank you so much for your participation. We’re very grateful to you for sharing your experiences.

*MyChart Non-Users*

1. As you might know, we are doing this study to learn more about how Trillium Health Partners can best serve patients and families. Trillium recently launched a tool called MyChart, which helps patients and families access their personal medical information from a computer or mobile device. Have you heard of MyChart?
   1. *If yes*, Can you tell me what you know about MyChart? How did you hear about it?
   2. *If no,* Do you have any questions about MyChart? Can I provide you more information about it to help give background information for this conversation?
2. The idea behind MyChart is to include patients in their own “circle of care,” which can help them participate in decision-making and receive information about their health quicker. Of course, it’s your choice to use MyChart or not, though. How would you describe your experience with receiving information about your health care THP? Do you have any suggestions for general improvements about managing or receiving updates about your health information?
3. Can you tell me your first thoughts when you hear[d] about MyChart?
4. Would you consider signing up for MyChart? Why or why not?
   1. Probe: *If yes*, what supports could Trillium Health Partners provide that would help you to sign up and use MyChart?
5. Are there specific things you feel are in the way of you accessing or using MyChart?
   1. Probes:
      1. Do you have access to the internet?
      2. Do you have a mobile device or computer?
      3. Are you comfortable using technology?
      4. Are you worried about the security or privacy of your health information on a portal like MyChart?
      5. Are you worried that you might not be able to use MyChart easily or understand the information you see on MyChart?
      6. MyChart is only offered in English right now. Would it be better for you if another language was available?
      7. Are there specific functions or features MyChart could offer that would interest you?
6. How important is it for you to have easy access to your personal health information and test results?
7. Is there a way that you prefer to receive reminders for upcoming appointments or prescription refills?
8. If they choose not to download MyChart to avoid test results:
   1. Are there specific aspects of receiving test results early that you find worrisome?
   2. Would you consider accessing MyChart if you could receive your results after your appointment? Or have the option to disable your results?
9. Have you had any negative experiences with similar online platforms in the past?
10. Do you feel that using the patient portal would add unnecessary complexity or inconvenience to your healthcare routine?
11. Is there anything else you’d like to tell me that we didn’t cover today?

Thank you so much for your participation. We’re very grateful to you for sharing your experiences.
